# Physical Fitness of School-Age Children after Cancer Treatment

**DOI:** 10.3390/ijerph16081436

**Published:** 2019-04-23

**Authors:** Iwona Malicka, Joanna Mrowiec, Natalia Sajkiewicz, Katarzyna Siewierska, Maria Czajkowska, Marek Woźniewski

**Affiliations:** 1 The Faculty of Physiotherapy, Wroclaw University School of Physical Education, 51-612 Wroclaw, Poland; mrowa171@vp.pl (J.M.); natalia.sajkiewicz@vp.pl (N.S.); katarzyna.siewierska@awf.wroc.pl (K.S.); marek.wozniewski@awf.wroc.pl (M.W.); 2 “Cape of Hope” Clinic of Bone Marrow Transplantation, Oncology and Hematology, Wroclaw University Clinical Hospital, 50-556 Wroclaw, Poland; maja_cz46@wp.pl

**Keywords:** child health, cancer, physical activity, physical fitness

## Abstract

The aim of the study was to assess physical fitness of school-age children after cancer treatment. The study was comprised of one hundred and fifty six children—children after cancer treatment (*n* = 71, mean age 11.22 ± 3.11 years) vs. healthy children previously untreated for cancer (*n* = 85, mean age 10.71 ± 1.22 years). Physical fitness was assessed indirectly based on a ball throw (assessment of strength, coordination, and upper limb speed), long jump (assessment of jumping ability, speed, and coordination), and a 60 m run (assessment of speed). The analysis was performed based on the Student’s t-test for independent samples and the analysis of variance and the post-hoc least significant difference test (LSD test). Children from the study group threw the ball closer and had shorter long jump performance compared to the control group, i.e., 12.93 [m] vs. 19.79 [m], respectively (*p* < 0.001) and 2.46 [m] vs. 2.70 [m], respectively (*p* = 0.02). However, their mean running time was longer, i.e., 13.33 [s] vs. 11.73 [s], respectively (*p* = 0.01). Division according to sex showed additionally significantly shorter ball throw distance in the study group in both girls (*p* = 0.001) and boys (*p* < 0.001), significantly shorter jump length in the group of girls (*p* = 0.01), and significantly longer running time in the group of boys (*p* = 0.04). Children treated for cancer are characterized by decreased physical fitness, and motor ability is sex-dependent. Both groups showed decreased strength, coordination, and upper limb speed. Additionally, worse jumping ability was found in girls whereas decreased speed was observed in boys.

## 1. Introduction

In recent years, an increase in the number of children cured of cancer has been observed due to the introduction of modern methods of treatment [1,2]. Currently, the 5 year relapse-free survival is 80% [2,3]. It is estimated that there are about 500,000 childhood cancer survivors in Europe. This number will probably increase to 1 million by the year 2025 [4]. This growing number of survivors, with many years of life ahead of them, has raised the necessity for knowledge related to the risks of adverse long-term sequelae of the life-saving treatments in order to provide optimal screening and care and to identify and provide adequate interventions [2].

The most common cancers in children include leukemias (34%), brain tumors (23%), and lymphomas (12%) [5]. To varying degrees, these patients experience a wide spectrum of long-term adverse health consequences of the life-saving treatments that can affect almost any organ or body system [2]. A high or severe burden of adverse events is observed in 55% of survivors who received radiotherapy only, in 25% of survivors who had surgery only, and in 15% of survivors treated with chemotherapy (the median attained age at the end of follow-up was 24.4 years) [6]. These complications are most commonly connected with the following systems: neurological (43%), circulatory and endocrine (18%), respiratory (11%), and musculoskeletal (10%) [7]. Therefore, complications result in a decrease in daily physical activity by about 60%, and also lead to overall lower physical fitness by 69% and physical capacity by 20% [8].

Of note, physical fitness also depends on ontogenesis and is slightly different in boys and girls. An increase in body height in girls is observed between 11 and 14 years of age, while in boys it is noted between 13 and 16 years of age. An increase in body mass in girls is reported between 14 and 15 years of age, unlike in boys in whom weight gain is found between 15 and 17 years of age. Motor features are also developed at a different age stage depending on sex. A physically fit child should achieve appropriate age-related results in terms of speed, jumping skills, muscular strength, and endurance. It is possible when individual systems stay in homeostasis [9].

Motor abilities are also trained through regular physical activity. Lack of regularity, too little or no further stimulating range of physical activity results in regression of motor abilities. Therefore, regular physical activity and physical effort are very crucial to children and are essential for their normal motor development. Healthy children and those treated for cancer need daily physical activity matched to their age, sex, and ability. Properly selected exercises or sports disciplines allow not only normal development of motor abilities, but also guarantee psychophysical well-being. Studies on children after cancer treatment indicate a linear relationship between physical activity and the quality of life and general fitness [10]. While the quality of life of these patients is the subject of numerous studies [11,12,13,14,15,16], the assessment of physical fitness primarily refers to supervised rehabilitation programs that are limited in time [17,18,19,20]. Daily physical activity, which is a lifestyle affecting general physical fitness, is a very important and still underestimated factor of both primary and secondary cancer prevention. 

Children undergoing cancer treatment usually do not undertake physical activity. A study of Kowaluk et al. showed that none of the children undergoing cancer treatment undertook physical activity that would last at least 60 minutes a day. Additionally, as many as 77% of children undergoing oncological treatment did not perform any physically effortful activities leading to general fatigue [21]. Similar results were presented by Aznar et al. [22], who also showed significantly lower levels of total weekly time of moderate to vigorous physical activity in children treated for leukemia. Götte et al. [23], in turn, paid attention to a significantly lower level of physical activity in all dimensions of daily activity, primarily in children with bone malignant tumors and those who were hospitalized. Children after cancer treatment are also less active than their peers. Considering the fact that they still have many years to live, it is important to pay attention to the levels of their physical activity and fitness, which determine their quality of life [24]. Therefore, it is crucial to implement specialist rehabilitation and systematic physical activity that are very important to regain physical fitness lost due to cancer treatment. Properly selected physical exercises and sport help to avoid cancer-related complications at a later stage [25].

The aim of the study was to assess physical fitness of school-age children after cancer treatment who did not participate in supervised rehabilitation programs.

## 2. Materials and Methods 

### 2.1. Study Group

One hundred and fifty six children (72 girls and 84 boys) were enrolled in the study.

The study group (1) consisted of 71 children after cancer treatment (30 girls—Subgroup 1 and 41 boys—Subgroup 2) who were the participants of the Lower Silesian Onco-Olympic Games of Children and Adolescents in Wroclaw, Poland. The diagnoses included acute lymphoblastic leukemia, acute myeloid leukemia, Hodgkin’s lymphoma, non-Hodgkin lymphoma (NHL-T i NHL-B), Evans syndrome, chronic myeloid leukemia, myelodysplastic syndrome, secondary myelodysplastic syndrome, MPS—myeloproliferative disease, ependymoma, anaplastic oligodendroglioma, osteosarcoma, Wilms’ tumor, nasopharyngeal cancer, and ovarian cancer. Chemotherapy (89.4%), radiotherapy (19.1%), transplantation (36.2%), and steroid and immunoglobulin treatment and surgery (8.5%) were used as forms of treatment. All children had undergone cancer treatment (the mean time since completion of treatment was 3.3 ± 2.4 years) and had physician consent for participation in the Lower Silesian Onco-Olympic Games of Children and Adolescents. The mean age of the subjects was 11.22 ± 3.11 years, the mean body height was 1.45 ± 0.17 [m], the mean body weight was 40.15 ± 14.66 [kg], and body mass index (BMI) was 18.72 ± 4.53 [kg/m^2^].

The control group (2) consisted of 85 healthy children (42 girls—Subgroup 3 and 43 boys—Subgroup 4) who were students of Primary School and Nursery Unit No. 18 in Wroclaw (mean age 10.71 ± 1.22 years). The mean body height was 1.42 ± 0.07 [m] and the mean body weight was 36.30 ± 7.74 [kg]. The mean BMI was calculated based on the above values and was 22.42 ± 5.85 [kg/m^2^]. 

Table 1 shows the characteristics of the four subgroups. 

The participants were inhabitants of Lower Silesia (Poland) who were elementary school students. Additionally, physician consent was required (study group). In the case of the control group, children were not medically exempt from physical education classes. We excluded the participants in supervised rehabilitation programs, those who practiced sports, and children with contraindications to physical exercises. Informed consent was obtained from parents or legal guardians of the participants prior to the competition.

### 2.2. Research Methods

Motor abilities were assessed. For this purpose, the results from three athletic competitions were considered, i.e., palant ball throw (assessment of strength, coordination, and upper limb speed), long jump (assessment of jumping ability, speed, and coordination), and a 60 m run (assessment of speed) [9]. As opposed to the medicine ball, the palant ball is a light ball that weighs about 150 g and is used in Poland for a game that is similar to baseball.

The 60 m run was performed on a track (low start position). The participants started the run from the starting line at the sound of the signal. The result obtained from the manual measurement was recorded with the accuracy of 0.01 s after crossing the finish line.

Long jump included a run-up (30 m). At the sign of the referee, the subjects accelerated. At the end of the run-up there was a zone (1 m) from which the takeoff took place. The participants had two trials and then three normal jumps, of which the best one was chosen. The distance between the first mark and the takeoff point was measured with a measuring tape with an accuracy of 0.01 m. 

A throw was performed without run-up with the use of a palant ball. Each child could have two trials, followed by three normal throws, of which the best one was selected. The field of throws was determined, and the distance markers were placed every 5 m. A measuring tape was placed perpendicular to the end line in the long axis of the run-up track. The throws were measured with an accuracy of 0.5 m from the designated throw line to the point where the ball fell. 

Physical fitness was assessed indirectly based on the analysis of the results obtained from three athletic competitions evaluating particular motor abilities. Motor abilities reflect the current state of the body to perform various types of motor tasks. In turn, physical fitness determines the ability to perform a variety of motor tasks dependent on the state of motor abilities, i.e., strength, speed, coordination, and endurance. These criteria are examined by subjecting children to fitness tests, including sprinting, long jump, and palant ball throw. Additionally, endurance running and medicine ball throw are also included for the complete assessment. However, they were excluded due to the health condition of children after cancer treatment and no physician consent.

In children after cancer treatment, the study was conducted during the Lower Silesian Onco-Olympic Games of Children and Adolescents, which is a sports event promoting physical effort in treatment, rehabilitation, and physical activity in patients affected by cancer. The aim of the Games is to overcome stereotypical perception about the unnecessary or even harmful nature of physical effort in cancer disease. The event is organized once a year for children after cancer treatment as part of Children’s Day during which they compete against each other in athletic competitions based on natural forms of movement, i.e., the 60 m run, palant ball throw, and long jump. Therefore, the assessment was made in natural conditions and the stress factor was not involved in the study. In the control group, the tests were performed during physical education classes. 

### 2.3. Ethics

The study was approved by the Local Bioethics Committee at the University of Physical Education in Wroclaw, Poland (consent no 7/2018).

### 2.4. Statistical Analysis

Statistica software 10.0 (StatSoft^®^, Cracow, Poland) was used for statistical calculations. Statistical means and standard deviations were calculated. The normality of distribution was verified with the Shapiro-Wilk test and the homogeneity of variance with the use of Levene’s test. Student’s t-test for independent samples was used (study group vs. control group) and the analysis of variance was performed using the post-hoc least significant difference test (LSD test) with the division into four groups according to sex (girls, boys) and cancer history (children after cancer treatment, healthy children). The level of statistical significance was adopted as *p* < 0.05. 

## 3. Results

**Figure 1:**
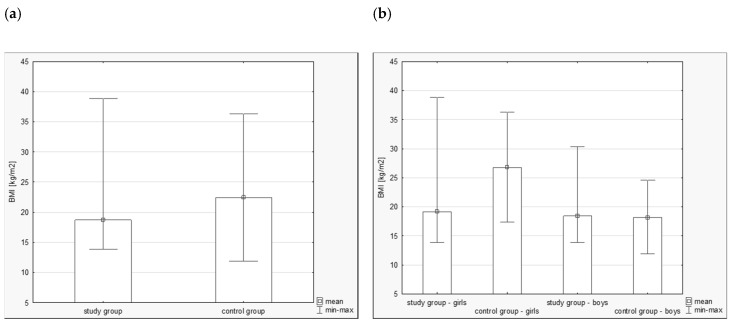
The mean body mass index—BMI [kg/m^2^] in the study groups. (**a**) study group vs. control group, (**b**) groups according to sex.

No differences were observed in terms of age, height, or body weight between groups 1 and 2. However, a statistically significant difference was found for BMI. In the study group, mean BMI was 18.72 [kg/m^2^] while in the control group it was 22.42 [kg/m^2^] (*p* < 0.001), as shown in Figure 1a. After additional differentiation of both groups according to sex, BMI reached the following values: for the group of girls after cancer treatment (Subgroup 1)—19.16 [kg/m^2^], for the group of healthy girls (Subgroup 2)—26.78 [kg/m^2^], for boys after cancer treatment (Subgroup 3)—18.40 [kg/m^2^], and for healthy boys (Subgroup 4)—18.17 [kg/m^2^]. The group of healthy girls was significantly different from the other three groups (*p* < 0.001), as shown in Figure 1b. 

**Figure 2:**
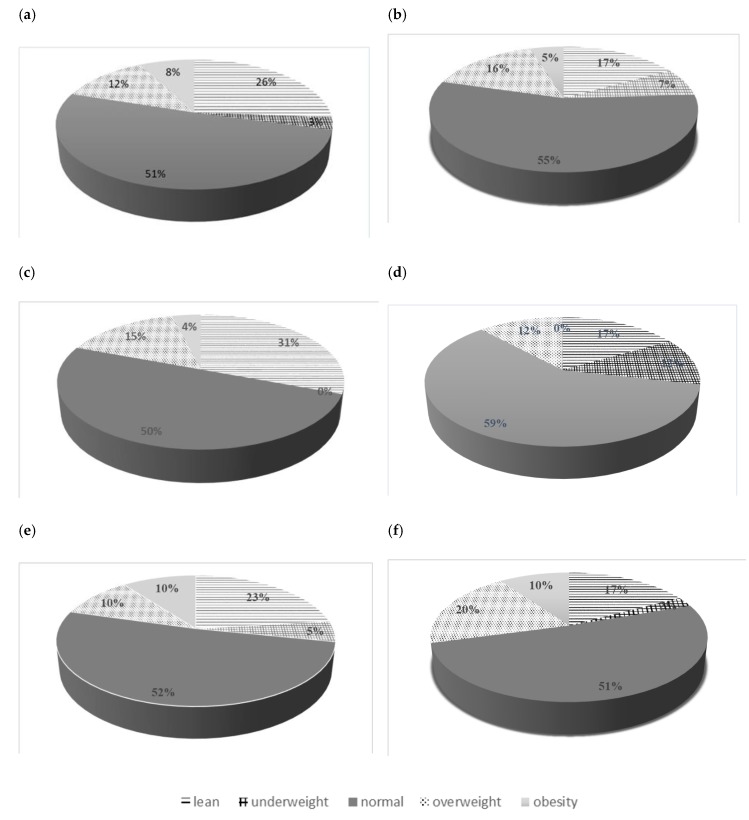
Analysis of the BMI in the groups: (**a**) study group, (**b**) control group, (**c**) girls from the study group, (**d**) girls from the control group, (**e**) boys from the study group, (**f**) boys from the control group.

Due to the fact that the study subjects were children, the BMI was additionally analyzed. The obtained results were compared with WHO growth charts. For the analysis of the obtained data, BMI < 5th percentile was adopted as the limit value for underweight. BMI between 5th and 85th percentile was considered normal weight, BMI between 85th and 95th was overweight and BMI > 95th percentile was considered obesity. No significant deviations from the norm were observed in over 50% of children, irrespective of the group. No statistically significant differences were found in the individual ranges of BMI. Detailed values are presented in Figure 2 a–f.

**Figure 3:**
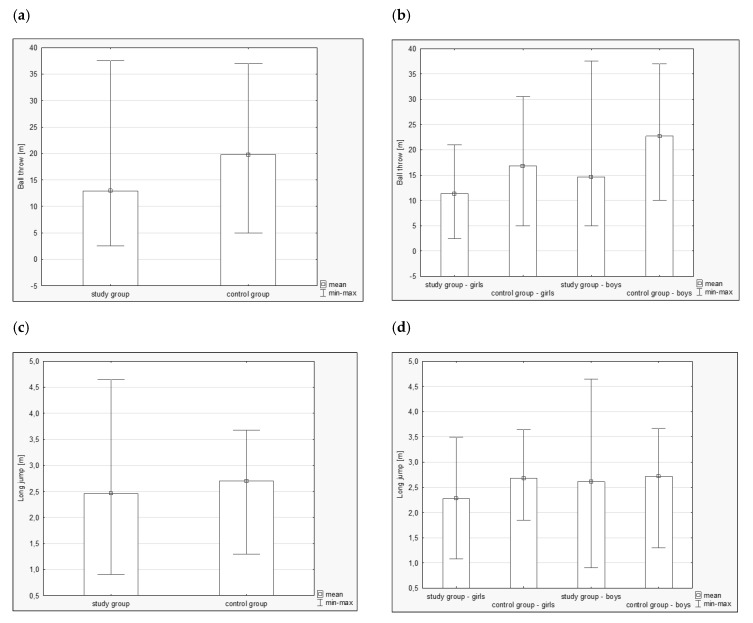

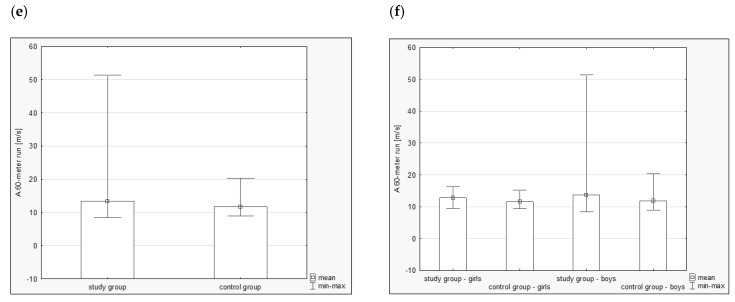
(**a**) Ball throw in the study groups. (**b**) Ball throw in the study groups according to sex. (**c**) Long jump in the study groups. (**d**) Long jump in the study groups according to sex. (**e**) A 60 m run in the study groups. (**f**) A 60 m run in the study groups according to sex.

A significant difference was observed in the palant ball throw between the study group and the control group, i.e., 12.93 [m] and 19.79 [m], respectively (*p* < 0.001), as shown in Figure 3a. Additionally, after the division of both groups according to sex, the following results were obtained: Subgroup 1—11.35 [m], Subgroup 2—16.81 [m], Subgroup 3—14.64 [m], and Subgroup 4—22.70 [m], as shown in Figure 3b. A significant difference was observed for both girls—Subgroup 1 vs. Subgroup 2 (*p* = 0.001)—and boys—Subgroup 3 vs. Subgroup 4 (*p* < 0.001)—as shown in Figure 3b. 

A statistically significant difference between the study groups was also observed in the mean length of the long jump, i.e., 2.46 [m] vs. 2.70 [m] (*p* = 0.02), as shown in Figure 3c. Additionally, after the division into four groups according to sex, a significant difference was found among girls, i.e., 2.28 [m] in Subgroup 1 vs. 2.61 [m] in Subgroup 2 (*p* = 0.01). No significant difference was observed in the group of boys, i.e., 2.61 [m] in Subgroup 3 vs. 2.71 [m] in Subgroup 4, as shown in Figure 3d.

The study group covered a distance of 60 m in 13.33 [s] and the control group in 11.73 [s]. The observed results were significantly different at *p* = 0.01, as shown in Figure 3e. After further division into subgroups according to sex, the following results of the runs were obtained: Subgroup 1—12.82 [s], Subgroup 2—11.62 [s], Subgroup 3—13.70 [s], and Subgroup 4—11.84 [s]. A statistically significant difference was found in the group of boys at *p* = 0.04, as shown in Figure 3f.

## 4. Discussion

Currently in pediatric cancer, three-phase rehabilitation programs are proposed, i.e., hospital stage, early post-hospital stage, and late post-hospital stage. In phase I rehabilitation for childhood cancer patients, the following are of paramount importance—increasing physical activity and avoidance of sedentary behavior with an emphasis on motor skill acquisition. Following patient discharge, in phase II rehabilitation (home-based exercise) a shift to aerobic, strength, flexibility, and motor skill refinement is the main goal and will require parental education and exercise supervision. Phase III (independent, unsupervised, or team sports) will require the involvement of parents, coaches, and regional specialists to successfully monitor the child’s athletic development [25]. Organized forms of physical activity focused on the possibilities of a given group are one of the ways of encouraging children after cancer treatment to physical activity.

The palant ball throw was a sport competition in which a group of healthy children achieved better results irrespective of sex. The boys from the control group obtained the longest ball throw. Proper coordination, strength, and upper limb speed are necessary to obtain the long ball throw. The last two elements form the so-called explosive strength. Among boys, the development of explosive strength is uneven, and its increase is observed between 8 and 10 years of age and between 12 to 15 years of age. In girls, however, a high increase in explosive strength occurs between the ages of 8 and 13 [26]. Due to the fact that the mean age of the subjects was 11 years, these features could probably not reach their peak values in the group of children treated for cancer, which in turn contributed to the occurrence of differences between groups, among both girls and boys. Nevertheless, it should be noted that despite significant differences in the length of the palant ball throw, the results of the participants of the Onco-Olympic Games were within the range of this discipline. 

A similar statistically significant difference was observed for the long jump between healthy children and their peers after cancer treatment. In this competition, however, the further analysis (after division into subgroups) showed that a significantly shorter jump length was observed only in girls treated for cancer. It is best to train jumping ability in girls between 9 and 12 years of age, while in boys it should be trained at a later stage, i.e., between 13 and 15 years of age [26]. Statistically significant differences observed only in girls may be associated with treatment and the impaired period of peak development of jumping ability in this age as a result of therapy. In addition, attention should also be paid to a significant increase in bone length in the female sex at this period. Ness et al. [27] and Zhou et al. [28] showed that cancer treatment negatively influences the development of the skeletal system. However, maturation of the skeletal system is observed in boys at a later age [26]. Nevertheless, results of the long jump for both groups were within the permissible limits. 

The last competition was the 60 m run due to which locomotor speed and agility can be assessed. Also, in this athletic discipline, the participants of Onco-Olympic Games obtained in total worse results compared to their healthy peers. A further analysis, however, showed significant differences only in the group of boys. The development of this motor feature in girls is completed at the age of 15, whereas in boys it lasts up to 18 years of age. In the period between 8 and 12 years of age, speed in both sexes is very similar. However, generally males have a higher level of this feature compared to females [26]. No differentiation in the results of the run in the group of girls may result from puberty observed in girls at 9–12 years of age, which is often associated with reluctance to daily physical activity, irrespective of health condition [29]. However, in the group of boys, the disease and its treatment most likely contributed to decreased physical activity and, as a result, to significant differences between groups. Deisenroth et al. [30] also observed lower muscle strength of the lower limbs in pediatric cancer patients, which directly results in decreased neuromuscular coordination and consequently may lead to reduced locomotor performance that is necessary to obtain good results in the 60 m run. 

To conclude, children after cancer treatment who are not covered by supervised rehabilitation programs are characterized by decreased physical fitness. Sex also affects individual motor features [31,32]. As a result, it is crucial to implement early rehabilitation and encourage young patients to undertake physical activity by providing them with access to physical activities focused on the interests of a given age group depending on sex. Simioni et al. [20] showed that properly selected physical activity in pediatric patients during and after cancer treatment results in a number of favorable changes. It positively influences musculoskeletal and neuromuscular systems, improves cardiovascular and respiratory parameters, increasing physical capacity and reducing metabolic disorders.

Withycombe et al. [33] showed a 7.7-fold increased risk of obesity in children undergoing cancer treatment. Loeffen et al. [34] observed malnutrition in pediatric cancer patients at both diagnosis and treatment. Initially, lower BMI was observed in the participants of the Lower Silesian Onco-Olympic Games of Children and Adolescents. However, no significant differences were observed after performing a detailed analysis between the groups. In both groups more than 50% of children were within the normal range. Obesity was found in about 9% of children from the study group and in about 5% of healthy children, whereas lean body structure was found in about 25% and 16.5%, respectively.

Of note, low physical activity is connected with the increasing prevalence of overweight and obesity already in the early stage of life, which is related to a greater risk of cardiometabolic complications in children after cancer treatment [35]. 

The present study has some strengths. It was conducted among children after cancer treatment who did not participate in supervised rehabilitation programs. Most studies in this domain usually assess the impact of particular rehabilitation programs and activities on physical fitness and quality of life [18,19,20,36,37]. However, our study shows the functional status of children who were not involved in the rehabilitation process despite severe treatment. Global initiatives directed at pediatric cancer care and control are urgently needed. In addition, we obtained the knowledge on the level of motor abilities and their differentiation depending on sex, which was possible after the analysis of the results obtained from athletic competitions. This knowledge can be useful in planning and undertaking rehabilitation. Although physical fitness was assessed indirectly during the sports competitions organized for children, the assessment was made in natural conditions and was not related to additional stress due to activities connected with fitness evaluation. 

## 5. Limitations

The limitation of this study is related to the lack of knowledge on the level of daily physical activity undertaken by both healthy children and the children after cancer treatment. The only exclusion criterion was practicing any sport. In the future, adding a physical activity questionnaire to the physical activity assessment should be considered. 

One of the limitations of the present study is lack of health-related quality of life assessment (HRQoL). In particular, it would be very interesting to measure quality-adjusted life year (QALY), which is a well-known measure based on preferences for HRQoL in cancer [38,39]. Therefore, further research is warranted in this area. Onco-Olympic Games is a cyclical event. For that reason, a further follow-up will be extended to assess the quality of life in this group of children.

Furthermore, the study group included children with different cancer diagnoses and treatment. It was, however, related to the nature of the study and the assessment of motor features that allow indirectly to assess physical fitness based on the results of three athletic competitions (i.e., 60 m run, long jump, and palant ball throw) during the Lower Silesian Onco-Olympic Games of Children and Adolescents.

## 6. Conclusions

Pediatric cancer patients are characterized by decreased physical fitness and motor ability is sex-dependent. Both groups showed decrease in strength, coordination, and upper limb speed. Additionally, worse jumping ability was found in girls whereas reduced speed was observed in boys. In connection with the above, it is necessary to implement long-term care of childhood cancer survivors. It should include participation of these children in rehabilitation and organized forms of physical activity with consideration given to the level of physical fitness of children after cancer treatment and the level of their motor abilities.

## Figures and Tables

**Table 1:** Characteristics of four subgroups.

Variables	Study Group (Mean ± SD)		Control Group (Mean ± SD)	
	Girls (*n* = 30)	Boys (*n* = 41)	Girls (*n* = 42)	Boys (*n* = 43)
Age [years]	11.57 ± 3.67	10.97 ± 2.70	10.70 ± 1.25	10.72 ± 1.20
Body height [m]	1.45 ± 0.18	1.45 ± 0.16	1.41 ± 0.07	1.43 ± 0.06
Body weight [kg]	40.74 ± 13.70	39.74 ± 15.45	35.02 ± 7.18	37.54 ± 8.13
BMI [kg/m²]	19.26 ± 4.86	18.40 ± 4.31	26.78 ± 4.76	18.17 ± 2.95
